# Death of M. Hourmann

**Published:** 1842-11-26

**Authors:** 


*Death of M. Hourmann.*—We have to announce, with regret, the death of M. Hourmann, physician to the Female Venereal Hospital at Paris. M. Hourmann was a young physician of very high promise, and had recently received, as the reward of his labour, the decoration of the Legion of Honor. About twelve months ago he inoculated his finger with venereal matter, while dressing a woman laboring under syphilis ; secondary symptoms set in ; the bones of the head were attacked, and M. Hourmann sunk under the disease. —*Ibid.*

